# Enhancement of lung gene delivery after aerosol: a new strategy using non-viral complexes with antibacterial properties

**DOI:** 10.1042/BSR20160618

**Published:** 2017-11-17

**Authors:** Angélique Mottais, Tony Le Gall, Yann Sibiril, Julian Ravel, Véronique Laurent, Frédérique d’Arbonneau, Tristan Montier

**Affiliations:** “Gene Transfer and Gene Therapy” Team, INSERM UMR 1078; IBSAM; Laboratoire de Génétique Moléculaire et Histocompatibilité, CHRU Brest; UFR Médecine et Sciences de la Santé, 22 avenue Camille Desmoulins, Brest 29238, France

**Keywords:** Gene delivery, multimodal therapy, Antibacterial properties, Aerosol, Lung diseases, Nano carriers

## Abstract

The pathophysiology of obstructive pulmonary diseases, such as cystic fibrosis (CF), leads to the development of chronic infections in the respiratory tract. Thus, the symptomatic management of the disease requires, in particular, repetitive antibiotherapy. Besides these antibacterial treatments, certain pathologies, such as CF or chronic obstructive pulmonary disease (COPD), require the intake of many drugs. This simultaneous absorption may lead to undesirable drug interactions. For example, Orkambi® (lumacaftor/Ivacaftor, Vertex), a pharmacological drug employed to treat F508del patients, cannot be used with antibiotics such as rifampicin or rifabutin (rifamycin family) which are necessary to treat Mycobacteriaceae. As far as gene therapy is concerned, bacteria and/or biofilm in the airways present an additional barrier for gene transfer. Thus, aerosol administration of nanoparticles have to overcome many obstacles before allowing cellular penetration of therapeutic compounds. This review focusses on the development of aerosol formulations adapted to the respiratory tract and its multiple barriers. Then, formulations that are currently used in clinical applications are summarized depending on the active molecule delivered. Finally, we focus on new therapeutic approaches to reduce possible drug interactions by transferring the antibacterial activity to the nanocarrier while ensuring the transfection efficiency.

## Introduction

Gene therapy is a therapeutic strategy based on gene transfer approaches. They allow the input of nucleic acid constructs inside eukaryotic cells in order to correct a genetic abnormality (e.g. hereditary genetic disorders) or to regulate the expression of genes (e.g. cancers application). In most cases, it is necessary to have a carrier capable of conveying these nucleic acids. In fact, nucleic acid constructs being anionic polymers, cannot, except in some specific cases, interact with the negatively charged plasma membranes. Synthetic vectors are amongst the existing gene transfer systems. In 1987, the first synthetic carrier (DOTMA: N-[1-(2,3-dioleyloxy) propyl]-N,N,N-trimethylammonium chloride), allowing the introduction of DNA into mammalian cells, was developed by Felgner and co-workers [1]. This family of carriers is now used in 4.6% of gene therapy clinical trials (http://www.wiley.com/legacy/wileychi/genmed/clinical/; the journal of gene medicine 2017). Unlike viral vectors, synthesis of chemical vectors is fully controlled and allows for mass production for high incidence pathologies. Moreover, they are, for the most part, neither immunogenic nor very cytotoxic [2,3]. This allows the re-administration of nucleic acid constructs, which is most often required since not only the DNA not integrate into the genome, but the expression of the transgene is a function of the lifetime of the transfected cell as well. Synthetic vectors are mainly cationic molecules that self-assemble with nucleic acids via electrostatic interactions that form polyplexes (polymers/nucleic acids) or lipoplexes (liposomes/nucleic acids) [3–6]. In addition to facilitating internalization in the eukaryotic cell, this encapsulation also makes it possible to protect nucleic acids from possible degradation (interaction or degradation by enzymes in the extra or intracellular environment).

Gene transfer systems based on cationic polymers are classified into four different families depending on the nature of the polymer (poly-L-lysine derivatives [7], derivatives of polyethyleneimine (PEI) [8], dendrimers [9], and chitosan [10]). Some other synthetic vectors are bio-inspired from phospholipids that form plasma membranes and are called cationic lipids [11]. These molecules of amphiphilic nature are composed of three parts: a polar head, a spacer, and a hydrophobic domain. Cationic lipids have been classified into four major subfamilies depending on the number of positive charges and the nature of the hydrophobic domain: monocationic, polycationic, cholesterol-derived monocationic, and cholesterol-derived polycationic. In order to improve transfection efficiency, numerous cationic lipids have been synthesized and many formulations have been derived. Phase IIb clinical trial conducted by the U.K. cystic fibrosis (CF) gene therapy consortium showed that the non-viral aerosolization gene therapy approach for CF application was beneficial and allowed CF patients to maintain their respiratory capabilities after an administration per month for a year (forced expiratory volume in 1 s (FEV1) + 3.7%) [12].

The intracellular barriers have been extensively studied in order to better understand how a gene transfer system should behave and know which essential properties are necessary for functional non-viral gene therapy, especially in the respiratory tract [13–16]. Nevertheless, extracellular barriers such as mucus, bacteria, and inflammation are important and decisive primary barriers which determine the extent of the contact between the gene transfer systems and the target cells [17–19]. The nature of bacterial flora in the pulmonary environment has not been taken into account in the evaluation of synthetic vectors nor in the clinical trial carried out by the British CF consortium [12]. Only pulmonary exacerbations were included as a clinical end point. However, some studies have shown that bacteria constitute an extracellular barrier that can oppose gene transfer [20,21]. If the airways do indeed seem to be the natural way to treat respiratory diseases, the effectiveness of the treatment has been slowed by the extra and intracellular barriers. This observation raises the question of both the mode of administration and the barriers faced by gene transfer. For example, the viral envelope of most recombinant vectors have difficulty withstanding the shear forces caused by an aerosol [22]. Then, for viruses still whole, their penetration into the hyperviscous mucus is difficult [23].

Currently, patients with pulmonary infections receive antibiotic therapy frequently. Taking any other treatment simultaneously, such as gene transfer, can create interactions and lead to a decrease in the expected beneficial effects. The new approach proposed in this review consists of developing formulations coupling simultaneously, the properties of gene transfer and the antibacterial effect. Meaning a single treatment will be administered in patients, decreasing the risks of drug interactions and increasing the therapeutic benefits. First, the anatomy of the airways, the mode of administration targetting these pathways, and their limits will be described. Then, the potential benefits of such an approach and the different formulations considered will be explained in terms of their clinical applications.

## Direct lung delivery

### The respiratory tract

**Figure 1:**
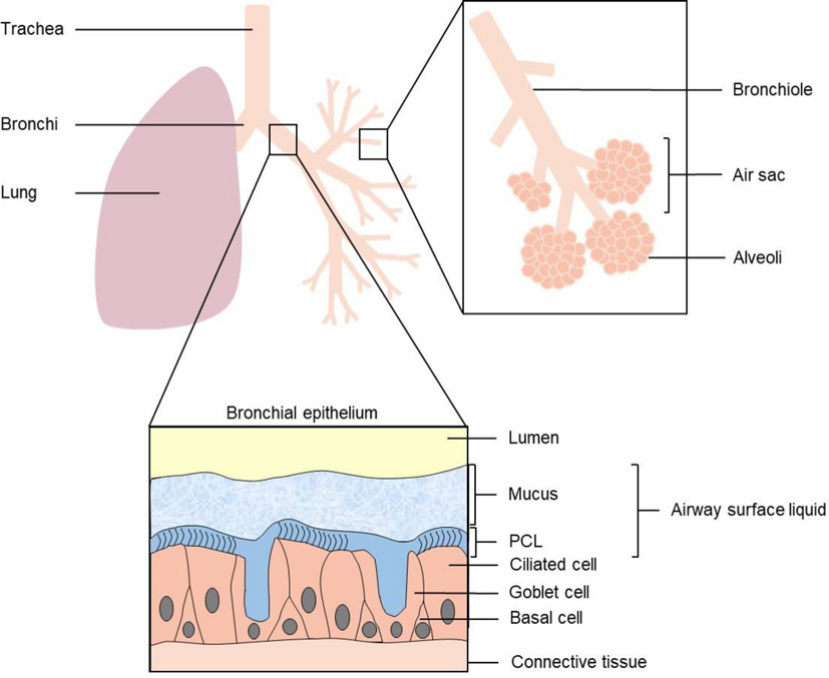
The organization and the structure of the respiratory tract Abbreviation: PCL, periciliary layer.

The respiratory tract consists of the upper airways (nasal and oral cavities, pharynx, and larynx) and lower airways (trachea, bronchi, and segmental bronchi). The upper airways allow the filtration, the heating, and the humidification of the incoming air. The primary role of the respiratory system is to ensure gas exchanges between the air and the blood. This exchange is performed at the pulmonary alveoli stage. The lower airways have a tree-like structure (Figure 1). The trachea, corresponding to the trunk (120–150 mm in length with a diameter of 14–15 mm), divides into two main bronchi (right bronchus: 2.5 cm in length for a diameter of 15 mm and left bronchus: 5 cm in length for 11 mm width), which themselves divide into segmental bronchi. Finally, the following bronchioles (diameter less than 1 mm) end with air sacs. The tracheopulmonary tree is divided into three distinct areas according to their function in the transport of oxygen. The conduction area extending from the trachea to the bronchioles allows the air to be conveyed. In this area, there is no air-to-blood gas exchange. Whereas the transition area corresponding to the bronchioles participates in the gas exchange. Finally, the respiratory area comprising all the pulmonary alveoli allows for most of the gas exchange by diffusion. The decrease in respiratory function is often due to repeated aggression on the respiratory tract. For example, in smokers or in chronic obstructive pulmonary disease (COPD) patients, the inhaled toxicants will gradually destroy the cellular layers, leading to a reduction in gas exchange giving rise to an increase in the partial pressure of oxygen. Similarly, in CF patients, repeated cycles of infection and inflammation will lead to fibrosis of the pulmonary parenchyma and a decrease in respiratory function (measured by FEV1).

The structure of the airway epithelium varies depending on the section. The bronchi are lined with a pseudostratified epithelium, whereas in the bronchioles, the epithelium is simple cylindrical and then cuboidal. The tracheobronchial epithelium (trachea and bronchi) is composed of ciliated cells permitting mucociliary clearance, globlet cells, and basal cells (Figure 1). The ciliated cells allow the elimination of pollutants trapped on the surface’s liquid which covers the epithelium. This surface liquid (‘airway surface liquid’ (ASL)) is composed of periciliary layer (PCL) and mucus layer. Compared with the mucus, the PCL has a low viscosity [24]. The mucus is composed of salts, proteins (glycoproteins, mucins, mucoproteins), and water [25]. It corresponds to the product of secretions from different cells (globlet cells in the trachea and clara cells in the bronchioles). The hydration state of the surface liquid is dependent on the ionic transports (chloride ions and sodium ions in part) [26,27]. Some pathologies, such as CF, induce a defect in regulation or expression of the channels involved in ion transport, resulting in dehydration of the surface liquid and a defect in mucociliary clearance [28]. This hyperviscous mucus becomes a favorable environment for microbial infections’ development.

### Targetting the respiratory tract by aerosolization

Aerosolization is currently the preferred mode of administration for airway targetting. This technique of administration is non-invasive and induces little stress for patients commonly treated with aerosol. For example, asthmatic patients inhale bronchodilators (terbutaline sulphate, salbutamol sulphate, ipratropium bromide) even before the age of 3 years. Aerosolization allows the passage of a liquid solution in the form of microdroplets. Several types of aerosolization systems exist: jet nebulizers, ultrasonic, or membrane nebulizers. Jet nebulization uses a compressed gas (air or oxygen) to generate microdroplets. With the ultrasonic system of nebulizers, the aerosol is formed by high-frequency vibration of a liquid. The microdroplets of the third type of aerosolization system are obtained after passage of the solution through a membrane. The size of the droplets formed varies according to the aerosolization system used [29]. The choice of the system is important because depositing the aerosol within the respiratory tract is defined by the size of the droplets formed [30]. Given the variable respiratory flow, the finest particles diffuse deeper at the alveolar level [31]. Three depositing mechanisms can be observed. The impaction phenomenon for droplets larger than 5 μm is due to the respiratory tract structure. Particles between 1 and 5 μm are sedimented, while droplets below 1 μm diffuse by Brownian motion in the bronchioles and the alveoli. The administered fluid volumes are very limited, same as the nasal instillation, because an excess of liquid can lead to drowning.

### Pharmacokinetics of nanocomplexes after aerosol delivery

**Figure 2:**
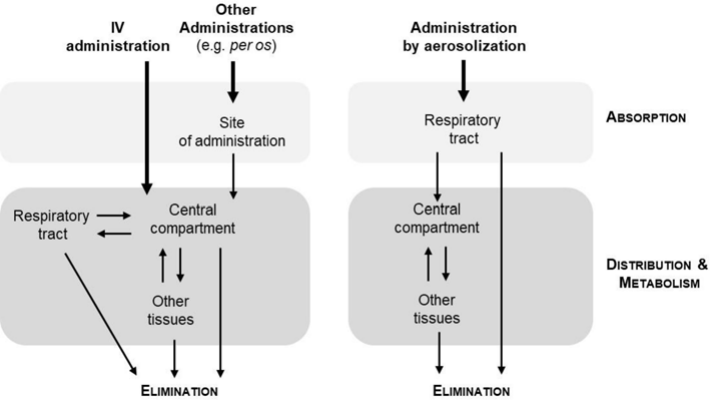
Pharmacokinetics according to the administration used The aerosolization allows direct targetting of the lungs and thus bypasses the blood circulation.

**Figure 3:**
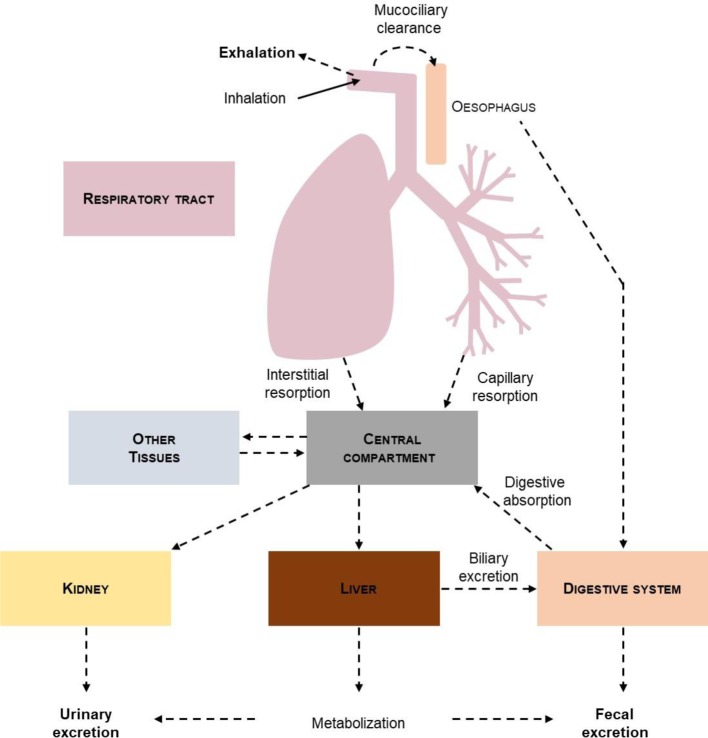
Elimination pathways of an inhaled drug Some of the inhaled drugs are eliminated by exhalation during breathing. The mucociliary clearance leading to the coughing up of sputum allows the more or less rapid elimination of the active ingredients. Once in the trachea, the active ingredients are swallowed and arrive in the digestive tract. Unlike oral administration, few drugs diffuse into the bloodstream due to the small quantity that reaches the pulmonary alveoli, which is the only point of passage to the blood (modified from [35]).

The major advantage of administration by inhalation is its pharmacological properties. Pharmacokinetics and pharmacodynamics indeed determine the therapeutic effect of a drug. Unlike the other major modes of administration, inhalation makes it possible to circumvent the blood circulation, and to avoid the first-pass effect of the liver which may lead to a reduction in the quantity of active principles reaching the targetting tissue and to potential side effects (Figure 2). For example, patients with type 1 Gaucher disease take an inhibitor of glocoyl ceramide synthase orally (eliglustat) [32]. To benefit from this treatment, a cytochrome P450 2D6 genotyping assay is required. The level of enzymatic activity of this cytochrome will determine the rate of metabolism of the drug and therefore the dose administered to the patient. This is also the case with certain classes of antibiotics. Amongst them, the aminoglycosides which bind the rRNAs (16S RNA) and thus block the translation of proteins are described as nephrotoxic and ototoxic agents when administered systemically [33]. The delivery of these antibiotics (example of gentamycin) by aerosolization has reduced these side effects [34]. The elimination of a drug also depends on the route of administration (Figure 3) [35]. If elimination does not occur fully, there are risks of accumulation which cause toxic side effects. When administered by inhalation, a large part of the active principle is eliminated by the mucociliary clearance and by exhalation. Nevertheless, the development of aerosolization systems has reduced the exhalation of treatments. The deposit site will determine the elimination kinetics [36]. The deeper the particles are deposited, the longer will be the elimination time.

### Physicochemical constraints due to aerosol protocol

**Figure 4:**
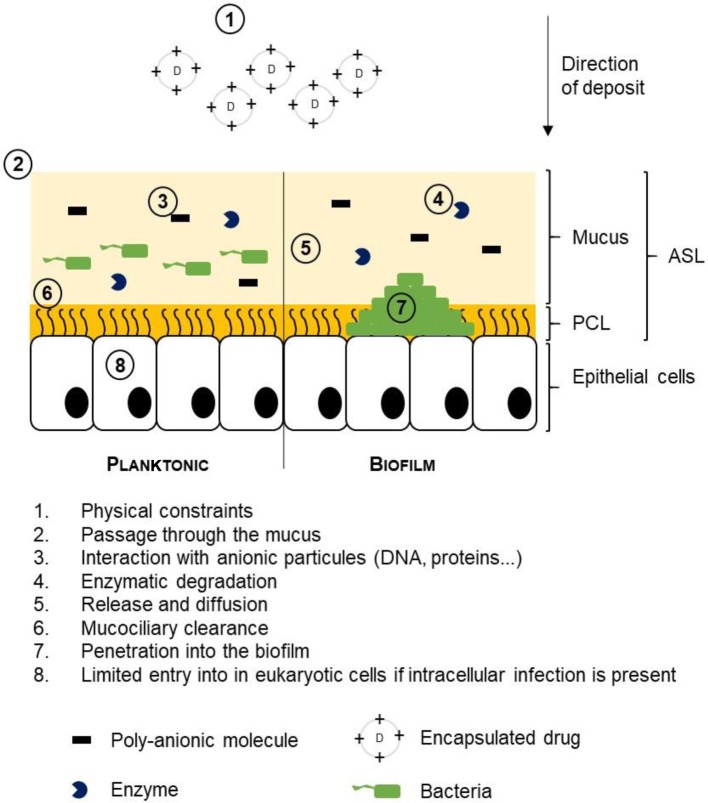
Extracellular factors limiting the therapeutic benefits of an aerosol Inhaled drugs encounter different physicochemical barriers that can negatively impact their activity. The aerosolization itself is very restrictive for use of drugs. It will determine the size and charge of the aerosolized particles and therefore the deposit site. To interact with eukaryotic cells, particles must penetrate a more or less viscous mucus and limit the interactions with the components and elements trapped in the mucus. ASL contains bacteria, in planktonic form or organized in a biofilm, which can release enzymes capable of degrading the active principle. In addition, bacteria in the form of a biofilm are protected by a very robust exopolyssacharide matrix. Once in contact with eukaryotic cells, the active ingredient must pass through the plasma membrane.

The physicochemical constraints associated with the aerosolization process are important. They may lead to a loss of the expected therapeutic effect (Figure 4). Not every active principle supports this mode of administration, hence the need for a formulation adapted to protect the active molecules. Moreover, depending on the nebulization system employed, the active principle will not react in the same way. For example, the dornase α, used to reduce viscosity of CF patient sputa, is degraded under the effect of heat when using an ultrasonic nebulizer [37]. Finally, research on the development of inhalation immunotherapy has shown that the antibodies poorly tolerate this mode of administration. An aggregation as well as chemical modifications were observed [38]. Once these physicochemical constraints have been overcome, the active principle must pass through various barriers mentioned below.

### Extracellular barriers encountered by nanoparticles in the respiratory tract

Treatments targetting the inside of epithelial cells encounter several obstacles during their transit from the upper respiratory tract to target cells. In addition to the mechanical movements of respiration, many extracellular barriers are present in the lungs and are a hindrance for inhalation treatments (Figure 4) [17,39].

The mucus is the first barrier whose role is to purify the air breathed in by the individual by trapping the inhaled particles. The thickness of non-pathological mucus is between 5 and 10 μm [40]. It is replaced every 10–20 min on an average [40]. Due to its composition and rheology, mucus is a key barrier against efficient inhaled therapy [19]. This filtering structure permits the passage of particles having a size of approximately 100–200 nm [41]. In some obstructive diseases such as CF, the mucus is more viscous and the mesh is tighter [42]. In addition to its high mucin concentration, the mucus contains many other anionic molecules such as cell debris, DNA, or actin. The latter, because of their charge, can interact with inhaled drugs and limit their activity [43]. For non-viral gene therapy, this leads to a distinct increase in negative charge concentration which will break apart the nucleic acids/vector complexes.

A second surface liquid called a pulmonary surfactant is also present on the inner surface of the pulmonary alveoli and facilitates respiratory movements. It is secreted by type II pneumocytes. It reduces the air/liquid surface tension on the alveoli facilitating respiration. It also plays a role in immune defense. It consists of 90% lipids (mostly dipalmitoylphosphatidylcholine) and 10% proteins [44]. This surfactant can trap active principles. In newborns, usually premature infants, a deficiency in pulmonary surfactant results in respiratory distress. Similarly in adults, frequent alterations of the pulmonary surfactant are observed. They can occur as a result of drowning and/or acute respiratory distress syndrome. Different exogenous surfactants exist and are administered endotracheally, which is quite invasive [45].

An innate defense system called mucociliary clearance helps eliminate inhaled toxicants (pollutants, microbes etc.) [46–48]. During inhalation, the particles are trapped in the mucus. The cilia present on the surface of the respiratory epithelium beat in a synchronized manner at a frequency of 1000–1500 beats per minute. This ciliary movement moves the mucus up to the trachea. The rate of upward movement of the mucus is between 5 and 20 mm/min. Once in the trachea, the mucus will be eliminated by the digestive tract or by expectoration. As previously stated, mucociliary clearance is a route of rapid elimination for inhaled drugs. It is therefore necessary that these therapeutical drugs must not remain blocked in the mucus which favors their elimination.

The development of high-throughput sequencing tools has demonstrated the presence of s pulmonary microbiota in the lower respiratory tract which had been long considered sterile, this includes healthy individuals [49]. This flora is present from an early age. It varies from one individual to another, depending on age and health status. Certain pathologies (CF, COPD, asthma) lead to an imbalance of this flora, favoring the progressive development of pathogens [50]. Bacteria responsible for lung infections produce enzymes capable of degrading certain drugs such as antibiotics.

The pulmonary microbiota comprises various bacterial species [49,51–53]. Bacteria can grow planktonically or as biofilm. The passage from planktonic bacteria to bacteria in biofilm leads to an increase in tolerance to treatments [54–56]. In the presence of a biofilm, the penetration of the active ingredient in the mucus is reduced due to the composition of the matrix formed, mainly of polysaccharides, proteins, nucleic acids, and lipids [57]. In a biofilm, the proximity of the bacteria and the presence of nucleic acids favor the dissemination of resistance by horizontal transmission of the genes. Finally, within a biofilm, part of the bacteria are dormant. This state of low active metabolism prevents the activity of certain antibacterial agents [58,59].

### Clinical applications for aerosol formulations

In France and most of western industrial countries, several drugs received marketing authorization for administration by aerosolization, their function are diverse: bronchodilators, corticosteroids, antibiotics, antiparasitic, anti-allergic, mucolytic, antiplatelet, and nasal decongestant (Table 1).

**Table 1:** Example of inhaled drugs

Pharmacological class	INN	Pathology	References
Bronchodilators	Ipratropium bromide	Asthma, COPD	[130]
	Terbutaline	Asthma, COPD	[131]
	Salbumatol	Asthma, COPD	[132]
Corticoids	Budesodine	Asthma	[133]
	Beclometasone	Asthma	[134]
Anti-infective agents	Tobramycin	CF	[135]
	Colistimethate sodium	CF	[136]
	Aztreonam	CF	[137]
	Pentamidine	Immunosuppressed	[138]
Mucolytics	Deoxyribonuclease 1	CF	[139]
Antiplatelet agent	Iloprost	PAH	[140]
Anti-allergic	Sodium cromoglycate	Asthma	[141]
Anesthesic	Lidocaine	Asthma	[142]

Abbreviations: INN, international non-proprietary name; PAH, pulmonary arterial hypertension.

Several pathologies benefit from this mode of administration. Currently, the causes of morbidity and mortality of patients with CF (or COPD) are lung damage. In these pathologies, aerosolization is partly used for the administration of antibiotics (Tobi®, Bramitob® Tobramycin; Cayston® Aztreonam lysine; Promixin® Colistimethate sodium; Tobi® Podhaler™ Tobramycin; Colobreathe® Colistimethate sodium; Aeroquin® Levofloxacin) but also for bronchodilators and mucolytics (Bronchitol® Mannitol, Pulmozyme® Dornase α) [37].

On the other hand, this route of administration has also been used in viral or non-viral gene therapy [12,60–64]. To date, clinical trials with viral vectors derived from adenovirus or associated adenovirus were disappointing and did not improve lung function. Alton and co-workers [12] conducted the first randomized clinical trial (phase IIb), as a double-blind (*n*=135), for non-viral gene therapy for CF (gene drug compared with placebo). The treatment was administered by aerosolization monthly for 1 year. A gain of 3.7% of the FEV1 was observed in treated patients. The results have been a proof of the feasibility of gene transfer by aerosolization with the absence of side effects.

## Transfecting formulations with antibacterial effects for gene therapy

### Potential benefits for gene delivery

**Figure 5:**
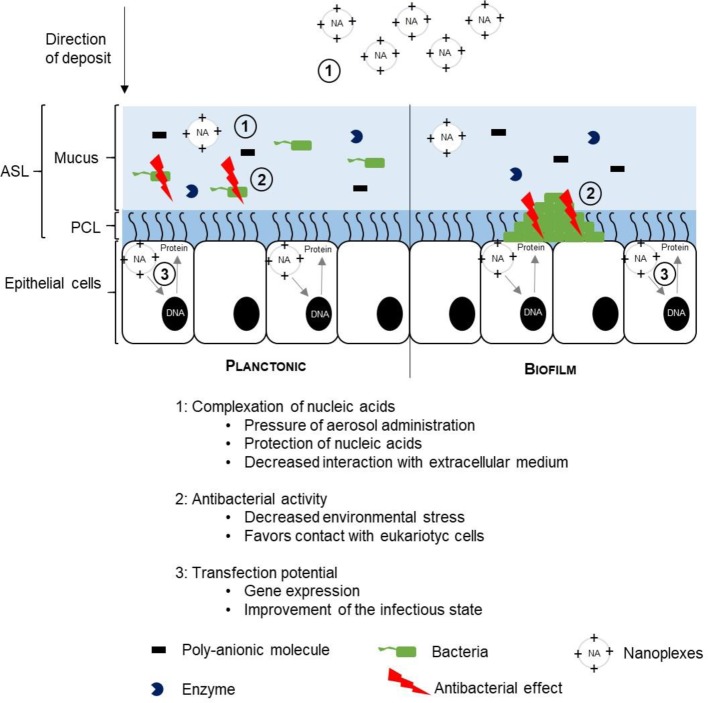
Multifunctional synthetic vectors: an advantage for gene transfer under infectious conditions The antibacterial activity of a gene transfer system would make it possible to transfect the eukaryotic cells in the presence of bacteria which impair the efficiency of the gene transfer. The production of toxins by bacteria and the induction of an inflammatory response leads to stress or even cell death. The antibacterial effect would eliminate the bacteria on the surface, promoting the transfection process and so the level of expression of the transgene.

The antibacterial activity of a gene transfer system could be beneficial for transfecting in an infected extracellular environment [20,65]. The benefits of such a combination of different activities are summarized in Figure 5.

Foremost, nucleic acids are fragile molecules that cannot tolerate aerosolization. The physicochemical constraints will induce a degradation of the nucleic acids which will be more or less important depending on their size [66,67]. The complexation of the nucleic acids by means of synthetic vectors is therefore required for this mode of administration. Moreover, this complexation will limit the degradation due to the presence of deoxyribonucleases in the extracellular matrix [21]. These enzymes are produced by bacteria such as *Staphylococcus aureus*. In an infected environment, the level of deoxyribonucleases is high. In addition, the formulations based on synthetic vectors are multimodular, which is to say that they can be adapted to the target environment by integrating several compounds enabling the possibility to cross the successive barriers. In example, pegylation (with PEG)) is a frequently used process to reduce surface charge and thus an easier penetration into the ASL [68]. Unlike the mucoadhesive agents which make it possible to increase the retention time of the active ingredient in the mucus, the PEG is a so-called mucopenetrating particle. A study on the delivery of an anti-inflammatory drug (dexamethasone) showed that PEG favored diffusion in the mucus and the release of the drug when compared with mucoadhesive particles such as poly(lactide-co-acid glycolide) (PLGA) [69].

Finally, the encapsulation is beneficial in order to potentiate the effects of the active molecules. Several active drugs (antibacterial, anticancer etc.) have been encapsulated by synthetic vectors. Alipour and co-workers [43] evaluated the antibacterial efficiency of two antibiotics (tobramycin or polymyxin B) encapsulated either by 1,2-dimyristoyl-sn-glycero-3-phosphocholine and cholesterol (DMPC:Chol, molar ratio: 1/2), or by 1,2-dipalmitoyl phosphatidylcholine and cholesterol (DPPC:Chol, molar ratio: 1/2). The antibacterial effect obtained on a strain of *Pseudomonas aeruginosa*, are far greater with the encapsulated form, even in the presence of polyanionic molecules (DNA, actin, lipopolysaccharides, lipoteichoic acids) frequently found in the sputum of CF patients. Similarly, Meers and co-workers [70] encapsulated amikacin (antibiotic) with a liposomal solution of DPPC:Chol (w/w: 2/1). They aerosolized the encapsulated formulation and the free form of the antibiotic in rats infected with *P. aeruginosa*. They found that the free form was ineffective in contrast with the encapsulated one. The lung concentration of bacteria in rat that benefited from the liposomal form was reduced. The aerosolization of the encapsulated form allowed a larger concentration of antibiotics to be present in the lungs for a longer amount of time, thereby limiting the emergence of bacterial resistance [70].

As we have seen, the presence of bacteria in the cellular environment can be harmful for gene transfer. This phenomenon can be accentuated in case of dysbiosis (an imbalance) of the bacterial flora leading to the appearance of infections. Growing bacteria is a very important barrier, which has long been neglected in the context of gene transfer applied to the respiratory tract. As mentioned above, bacteria produce toxins that can induce stress and/or cell death [71,72]. In addition, the infections are accompanied by strong inflammation. Inflammation induces the formation of reactive oxygen species which can lead to cell death [73]. All this will also contribute to limiting the expression of the transgene. The antibacterial activity of a transfecting formulation would eliminate bacteria localized in the cellular environment. Not only this elimination would promote access to eukaryotic cells in the presence of biofilm, it would also decrease the stress induced by the presence of bacteria in the cellular environment. Therefore, gene transfer could be carried out in a more favorable environment. Finally, expression of the transgene could further promote the eradication of infections. This is the case for CF, where the restoration of CFTR expression would induce a reduction in the risk of infection by the progressive fluidification of mucus through the restoration of ionic transports [74–77].

The aim of our strategy is to transfer the antibacterial effect of the active principle to the vector itself so as to be able to transport other active principles (such as nucleic acids for example), which in parallel reduces the risks of side effects generated by drug interactions. In order to obtain formulations with antibacterial and transfecting properties, several options are available. Several antibiotics have been encapsulated by synthetic vectors which have shown transfection capability. However, the antibiotics, which usually find themselves stuck with the complexes, need to be released to be efficient. The combination of antibacterial molecules and nucleic acids encapsulated by synthetic vectors could allow the two activities to be obtained. In addition, some synthetic vectors, which will be described hereinafter, are endowed with their own antibacterial activity. This activity would make it possible not to use the antibiotics which can cause an appearance of bacterial resistance.

### Antibiotics encapsulated by synthetic vectors

Attempts have been made to encapsulate antibiotics in order to decrease side effects since the 1980s [78,79]. Due to their fusion capability with plasma membranes and their capability of encapsulation, the synthetic vectors derived mainly from natural phospholipids have been used. These combinations have allowed the production of original formulations which will be described hereinafter.

Currently, two formulations of encapsulated antibiotics are used in Europe for pulmonary infections in aerosol delivery: TOBI® Podhaler™ (Novartis) and Arikace™ (Insmed) (Table 2). TOBI® Podhaler™ corresponds to the encapsulation of tobramycin (aminoside) by a cationic lipid, distearoyl phosphatidylcholine (DSPC). DSPC is used as a co-lipid for the formation of lipoplexes in the context of the delivery of interfering RNA [3,80,81]. This drug is prescribed for treating chronic pulmonary infections with *P. aeruginosa* in CF patients. Arikace™ is an aminoglycoside (amikacin) encapsulated by DPPC and cholesterol. DPPC is a natural lipid commonly used in gene transfer and has shown notable effects toward many different cell types but not for gene therapy in itself [82]. This drug has not received marketing authorization but is used clinically in a regulated context thanks to a transitional exemption. This encapsulated antibiotic can be delivered by aerosolization (eFlow® nebulizer) to CF patients whose pulmonary pathways are chronically infected with *P. aeruginosa* [70,83].

**Table 2:** Synthetic vectors used to encapsulate antibiotics

Encapsulated drug	Synthetic vectors chemical structure	References
**Tobramycin**	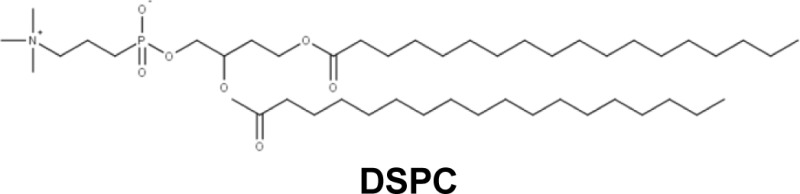	[81,143]
**Amikacin**	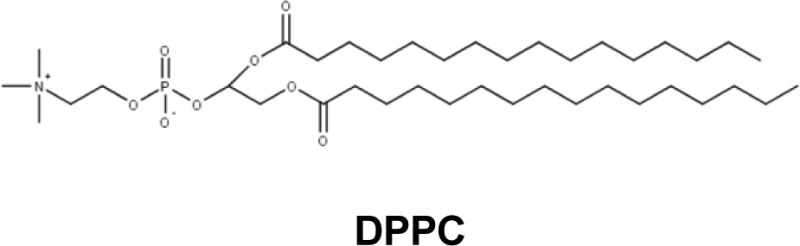	[8]

Other anti-infective agents are being evaluated clinically. Polymyxin is a polycationic antibiotic used to control Gram-negative infections. Its systemic administration induces significant side effects (nephrotoxicity, ototoxicity, and neuromuscular blockage). Several studies are searching for ways to encapsulate this antibiotic to limit its side effects and administer it by inhalation. The encapsulation of polymyxin B with DPPC showed better activity compared with the non-encapsulated form on a mouse pneumonia model and limits side effects [84–86].

Pulmaquin™ and Lipoquin™ (Aradigm, Hayward, CA, U.S.A.) are the encapsulated forms of ciprofloxacin with 65.9 mg/ml of hydrogenated phosphatidylcholine (HSPC) and 27 mg/ml of cholesterol. These two forms of liposomal antibiotics are used to treat chronic *P. aeruginosa* infections in immunocompromised patients. Lipoquin™ is also prescribed for CF patients and is administered by aerosol with a jet nebulizer. The release kinetics of ciprofloxacin varies according to the formulation. However, the release of the antibiotic is slower with Pulmaquin™ [87].

### Synthetic vectors with antibacterial effects

Some synthetic vectors are endowed with antibacterial and transfecting activities (Table 3).

**Table 3:** Example of synthetic vectors with an antibacterial activity

Synthetic vector family	Chemical structure	Antibacterial effect	References
**Antimicrobial peptide**	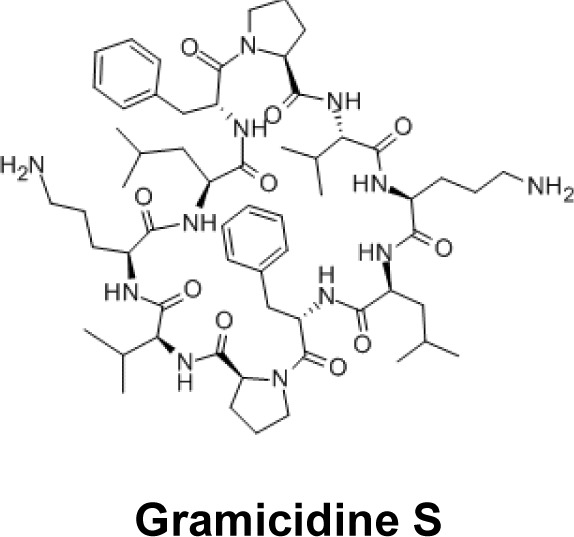	Gram+ Gram–	[88]
**Polymer**	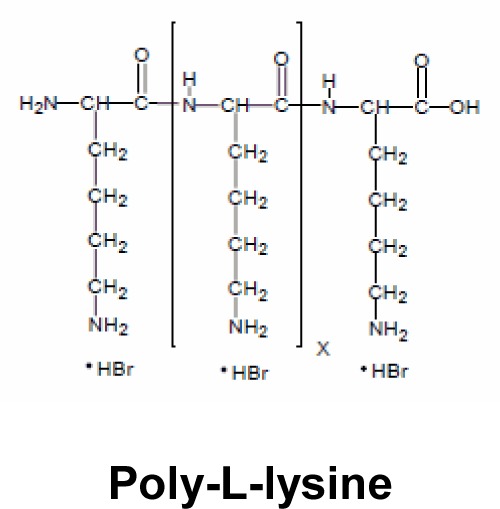	Gram+ Gram–	[124]
**Aminoside derivative**	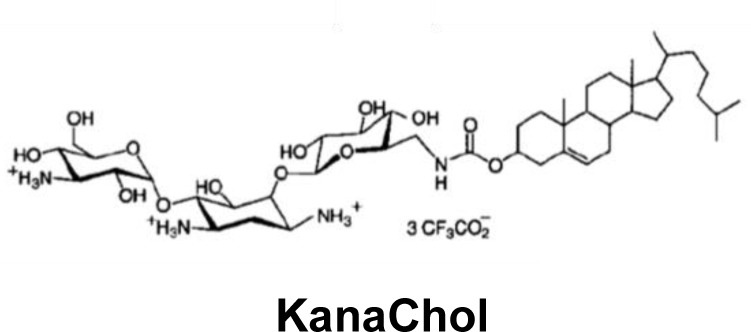	Gram+	[98–101]
**Sterol derivative cationic lipid**	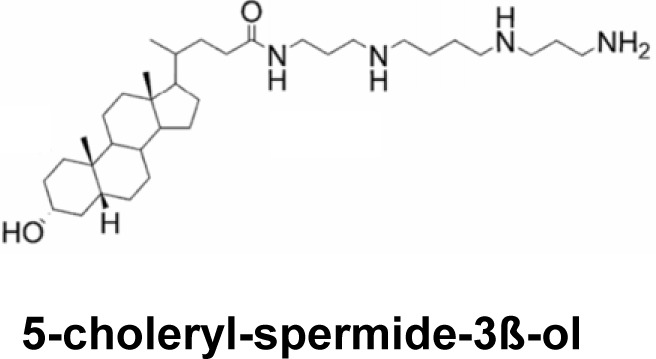	Gram+ Gram–	[20,115]
**Lipophosphoramidate**	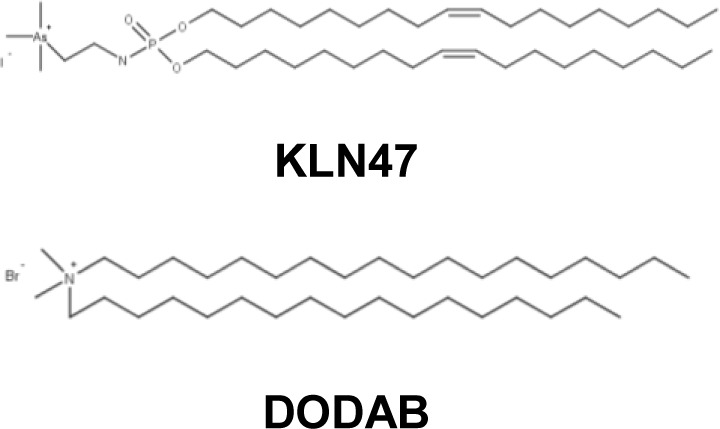	Gram+	[21]

### Antimicrobial peptides

Legendre and Szoka showed that antimicrobial peptides (gramicidin S, tyrocidin) with proved antibacterial activity, had a transfecting capability similar to that observed with cationic lipids [88]. These compounds have the ability to bind DNA by electrostatic interactions. Moreover, the fact that they are amphiphilic makes it possible to permeabilize the membranes [89,90]. These antimicrobial peptides act on Gram(+) and Gram(–) bacteria. The complexation of nucleic acids by antimicrobial peptides does not affect their antibacterial activity [91]. Their broad spectrum of activity makes antimicrobial peptides good candidates for antibacterial and transfecting formulations.

### Cationics lipids inspired from antibiotics

Some families of antibiotics such as aminoglycosides have the ability to bind nucleic acids (DNA and RNA) [92]. This observed characteristic, essential to gene transfer agents for correct complexation, was used for the synthesis of novel cationic lipids. Some lipid derivatives’ polar heads incorporate an aminoside such as kanamycin (KanaChol) [93,94] or neamine [95], a neomycin fragment. The aminoglycoside polar head makes it possible to condense the nucleic acid constructs and their cholesteryl motif facilitates the entry into the eukaryotic cell. After evaluation of their transfecting activity post-deposit, these lipid derivatives have shown an interesting efficiency for gene transfer in various mammalian cell lines [94,96,97]. In parallel, these amphiphilic derivatives of antibiotics also exhibit an antibacterial activity on *P. aeruginosa* [98–101].

### Cationic polymers

On one hand, Wu and Hsu evaluated the cationic polymers’ (water-based cationic polyurethanes) antibacterial potency on *Escherichia coli* and *S. aureus* strains which has proved to be potent [102]. On the other hand, high transfection efficiencies were obtained on a renal cell line [102].

Poly-L-lysine is a polymer commonly used for gene transfer [5,68,103,104]. In 2013, Dubois and co-workers [105] studied the antibacterial activity of poly-L-lysine. They found that this polymer made it possible to kill the bacteria such as the *P. aeruginosa* and *S. aureus* species which are frequently isolated from sputum of CF patients [106].

Finally, PEI and its branched or histidinylated derivatives are heavily used for gene transfer. They showed a very good transfection activity [5,107,108] on post-aerosolization on murine and sheep models as well [109–112]. In addition to this gene transfer capability, this family of synthetic vectors exhibited antibacterial (Gram(+) and Gram(–)) and antifungal activity post-deposit [113,114]. No studies evaluated the antibacterial potency of post-aerosolization PEI.

### Monocationic lipids derived from phospholipids

Similarly, other molecules not derived from antibiotics and which bind to DNA, such as spermine, have also shown transfection activity as well as an antibacterial effect on Gram(+) bacteria (*Bacillus subtilis*) and Gram (–) bacteria (*E. coli*) [115].

Fein and co-workers [20] are interested in the antibacterial and transfection properties of two steroid-derived cationic lipids called dexamethasone spermine (DS) and disubstituted spermine (D2S). These two compounds have been studied individually and as co-formulation. The evaluation of the various lipoplexes by direct deposit in the extracellular medium, revealed a good transfection activity on cell line A549 (epithelial cells derived from pulmonary carcinoma). Antimicrobial activity on Gram(–) bacteria (*E. coli* MG1655 and *P. aeruginosa* PAO1) and Gram(+) *B. subtilis* was obtained with D2S at low concentrations (5 μM). Given the chemical structure of D2S, they hypothesize that the antibacterial activity is due to the amphiphilic structure resembling antimicrobial peptides such as cathelicidin LL-37 which would favor destabilization of the bacterial membrane [20].

Subsequently, novel cationic derivatives of steroids containing other glucocorticoids (flumetasone, budesonide, and beclometasone) have been developed. Anti-inflammatory, antibacterial, transfectant, and cytotoxic activities were then evaluated [116]. These compounds showed antibacterial effects (a few µM depending on strain) on different strains of *P. aeruginosa* and methycillin-resistant *S. aureus* (Xen30). The transfection capability was evaluated in parallel by direct deposit on BAECs and A549 cell lines. Some compounds have levels of transfection similar to those obtained with Lipofectamine® 2000, a commercial transfer agent which has no antibacterial effect (Thermo Fischer Scientific).

Dodecyltrimethylammonium bromide (DODAB) can complex the DNA and thus allow the gene transfer [117,118]. Different studies have shown that quaternary ammonium compounds have antibacterial and antifungal activities [119–121]. Some geminis with two quaternary ammonium heads have shown good transfection efficiency due to their strong DNA interaction [122,123]. In addition, they exhibit antibacterial activity on Gram(+) bacteria (*E. coli* and *P. aeruginosa*) [124].

In 2013, our study confirmed the presence of antibacterial activity on some synthetic vectors originally designed for gene transfer [21]. After structure-activity analysis of a series of cationic lipophosphoramidate, it has been found that the nature of the polar head and aliphatic chains are the key elements of antibacterial potency. Contrary to trimethylammonium lipophosphoramidates, only a few cationic lipids with a trimethylarsonium or trimethylphosphonium polar head exhibit an antibacterial activity on different strains of *S. aureus*. The best antibacterial activity was obtained with arseno compounds. Furthermore, the structure and length of the aliphatic chains would affect the antibacterial activity. The degree of unsaturation and the length of the aliphatic chain permit the improvement of the antibacterial activity of trimethylarsonium lipophosphoramidate. This antibacterial activity was observed for relatively low concentrations, which are close to those used for the transfection of eukaryotic cells. This is an important point to simultaneously study both activities. To explore this hypothesis, liquid co-cultures of bacteria and human bronchial epithelial cells were used. In the present study, it was shown that the antibacterial activity of the cationic lipid makes it possible to obtain, in the presence of a bacterial infection, a transfection activity equivalent to that observed in the absence of bacteria.

## Synthetic vector based formulations

To broaden the spectrum of activity, previously studied silver compounds [125] were introduced into the formulation and experiments showed that the antibacterial activity was extended to Gram(–), which are problematic in CF, and that this activity was retained post-aerosolization (currently being submitted). To our knowledge, only one other study has combined molecules of different activities in order to obtain multifunctional transfer systems. Peng and co-workers [126] combined a gold nanoparticle (AuP) with an antimicrobial peptide (PEP ‘peptide sequence from lactoferrin’). This combination allowed Peng and co-workers [126] to efficiently transfect mesenchymal stem cells. The gold nanoparticles are known for their transfection power [127] as well as for their antibacterial activity on Gram(+) and Gram(–) bacteria [128,129]. However, these activities have not been tested by aerosolization.

## Conclusion

Antibiotics encapsulated in a formulation must be released in order to be available and come into contact with bacteria. Transferring the antibacterial effect directly to the vector would allow a more immediate effect. Multimodular vectors are a major asset to overcome the different barriers encountered and to act according to therapeutic targets, which are, not only the bacteria for the antibacterial effect at the extracellular level, but also the nucleus for the gene transfer. Besides gene transfer, many other applications such as administration of anticancer drugs, anti-inflammatory reagents, or various other molecules such as insulin can be considered. The formulations will be adapted to the constraints related to the inhaled administration and its environment. Finally, in order to combat the rapid increase in bacterial resistance, the antibacterial activity of the vector coupled with the antibacterial activity of the encapsulated antibiotic would allow the introduction of bi-antibiotic therapy. Thus, the targets will be more numerous and will allow the better treatment of the infections that are still difficult to treat today, such as nosocomial diseases which can infect immunosuppressed patients.

## Abbreviations

ASL: airway surface liquid
BAEc: Bovine Aortic Endothelial cells
CF: cystic fibrosis
CFTR: cystic Fibrosis Transmembrane conductane Regulator
COPD: chronic obstructive pulmonary disease
DPPC: 1,2-dipalmitoyl phosphatidylcholine
DSPC: distearoyl phosphatidylcholine
D2S: disubstituted spermine
LL-37: cathelcidin antimicrobial peptide
FEV1: forced expiratory volume in 1 s
F508del: deletion of three nucleotides (position 508) of cftr gene
PCL: periciliary layer
PEI: polyethyleneimine
PEP: Peptide sequence from lactoferrin
